# EEG microstates of dreams

**DOI:** 10.1038/s41598-020-74075-z

**Published:** 2020-10-13

**Authors:** Lucie Bréchet, Denis Brunet, Lampros Perogamvros, Giulio Tononi, Christoph M. Michel

**Affiliations:** 1 Functional Brain Mapping Laboratory, Fundamental Neuroscience Department, University Geneva, Campus Biotech, 9 Chemin des Mines, 1211 Geneva, Switzerland; 2 Biomedical Imaging Research Center (CIBM), Lausanne, Geneva, Switzerland; 3 Sleep and Cognition Neuroimaging Laboratory, Fundamental Neuroscience Department, University Geneva, Geneva, Switzerland; 4 Division of Pneumology, Department of Medicine, Geneva University Hospitals, Rue Gabrielle-Perret Gentil 4, 1205 Geneva, Switzerland; 5 Wisconsin Institute for Sleep and Consciousness, Department of Psychiatry, University of Wisconsin-Madison, 6001 Research Park Blvd, Madison, WI USA

**Keywords:** Neuroscience, Circadian rhythms and sleep

## Abstract

Why do people sometimes report that they remember dreams, while at other times they recall no experience? Despite the interest in dreams that may happen during the night, it has remained unclear which brain states determine whether these conscious experiences will occur and what prevents us from waking up during these episodes. Here we address this issue by comparing the EEG activity preceding awakenings with recalled vs. no recall of dreams using the EEG microstate approach. This approach characterizes transiently stable brain states of sub-second duration that involve neural networks with nearly synchronous dynamics. We found that two microstates (3 and 4) dominated during NREM sleep compared to resting wake. Further, within NREM sleep, microstate 3 was more expressed during periods followed by dream recall, whereas microstate 4 was less expressed. Source localization showed that microstate 3 encompassed the medial frontal lobe, whereas microstate 4 involved the occipital cortex, as well as thalamic and brainstem structures. Since NREM sleep is characterized by low-frequency synchronization, indicative of neuronal bistability, we interpret the increased presence of the “frontal” microstate 3 as a sign of deeper local deactivation, and the reduced presence of the “occipital” microstate 4 as a sign of local activation. The latter may account for the occurrence of dreaming with rich perceptual content, while the former may account for why the dreaming brain may undergo executive disconnection and remain asleep. This study demonstrates that NREM sleep consists of alternating brain states whose temporal dynamics determine whether conscious experience arises.

## Introduction

A distinct feature of Non Rapid Eye Movement (NREM) sleep is low-frequency (< 4 Hz, delta) EEG synchronization with frequent appearance of large oscillations (slow waves) that are associated with brief periods of neuronal hyperpolarization^1^. The switch between up- and down-states reflects the bistability of the membrane potential of cortical neurons during sleep, which underlies an impairment of causal interactions among brain networks and loss of neuronal integration and communication^2–4^. Single neuron physiology and modeling suggest that low-frequency activity is related to neural mechanisms of inhibition^5,6^.

However, despite the generally decreased neuronal activity during NREM sleep, there is clear evidence that also during NREM sleep, conscious experiences may appear in the form of dreams^7–10^. Some EEG studies demonstrated a link between successful dream recall in NREM sleep and lower temporal alpha power (8–12 Hz)^11,12^, lower frontal delta power (1–4 Hz)^13^ or lower global slow oscillations (< 1 Hz)^14^. Lesion studies have also indicated that posterior cortical regions (mainly occipital and parietal) are necessary for dreaming^15^. More recent studies using high-density EEG with 256 channels^16,17^ showed that low-frequency power, as well as slow-waves, decrease in parietal-occipital brain areas when participants reported experiencing dreams in NREM and REM sleep, independently of remembering their content or not. These results suggest that reduced neuronal bistability in posterior brain regions may restore effective neuronal interactions that support the occurrence of dreaming experiences. Intriguingly, the occurrence of dreams and posterior cortical activation usually does not lead to spontaneous awakenings, raising the question of which mechanisms may protect the continuation of sleep.

Besides the decrease of low-frequency power and slow waves in the posterior “hot-zone”, Siclari et al.^17^ also reported an increase of high-amplitude slow waves in frontal regions when participants remembered the content of their dreams. The occurrence of these frontal slow-waves was negatively correlated with the occurrence of posterior slow waves. Thus, frontal slow waves, reflecting increased bistability and deactivation of executive circuits, may be “protective” of sleep at times when the posterior cortico-thalamic system is activated, i.e. during dreaming. Accordingly, “partially distinct micro-states of the sleeping brain”^18^ may serve different functional roles in dreaming: one promoting dreams and the other protecting the brain from awakening.

A well-established way of identifying microstates in the ongoing EEG represents the clustering of the scalp electric field into dominant map topographies and fitting these cluster maps to the original data^19–21^. This approach has repeatedly shown that the EEG can be parceled into a sequence of stable brain states, each lasting around 50–100 ms. It has been shown that only a few (< 10) prototypical map topographies exist in the human brain at rest and that they reflect short-lasting synchronous activity of large-scale brain networks^22^. While the spatial configurations of these microstates remain uniform, many studies demonstrated that their temporal presence (such as explained, variance, occurrence, durations, transitions, etc.) are highly sensitive to distinct levels of consciousness, different neuropsychiatric diseases and cognitive contents (for reviews see Refs.^22,23^).

The one existing study on EEG microstates during different sleep stages^24^ showed that the sleeping brain expresses the same prototypical maps configurations as the awake brain, despite marked changes in the frequency content of the EEG. Notably, fMRI studies also show remarkable consistency between large-scale functional connectivity networks identified during resting wake and sleep, supporting the proposal that EEG microstate maps may represent the electrophysiological counterparts of fMRI resting state networks.

However, given the well-established role of low-frequency oscillations to prevent neuronal integration and communication discussed above, we here suggest that the presence of the microstates during NREM sleep (dominated by low-frequency activity) reflects a temporary process of suppression of functional integration between the nodes of the corresponding network. Consequently, we hypothesize that microstate maps that dominate during NREM sleep compared to resting wakefulness reflect those networks that are particularly important to be deactivated during sleep. On the other hand, given the finding of reduced low-frequency power in posterior regions during dreaming^16^ we hypothesized that microstate maps reflecting this network would be less inhibited from functional integration and would thus occur less often during episodes of dream recall vs. no recall.

In the present study we asked, first, whether the presence of prototypical microstates change between wake and NREM sleep and second, whether there are differences in the temporal characteristics of the microstates between dreaming and non-dreaming during NREM sleep. We also evaluated the spectral correlates of each of the microstates during the different conditions. We focused on awakenings from N2 sleep since the number of dream experiences, which are present or absent in this stage, are well balanced (compared to REM sleep, where most awakenings yield dream reports)^9,16^. Finally, we used source-modeling to localize the relevant microstates.

## Results

### EEG microstates during NREM sleep vs. resting wakefulness

**Figure 1:**
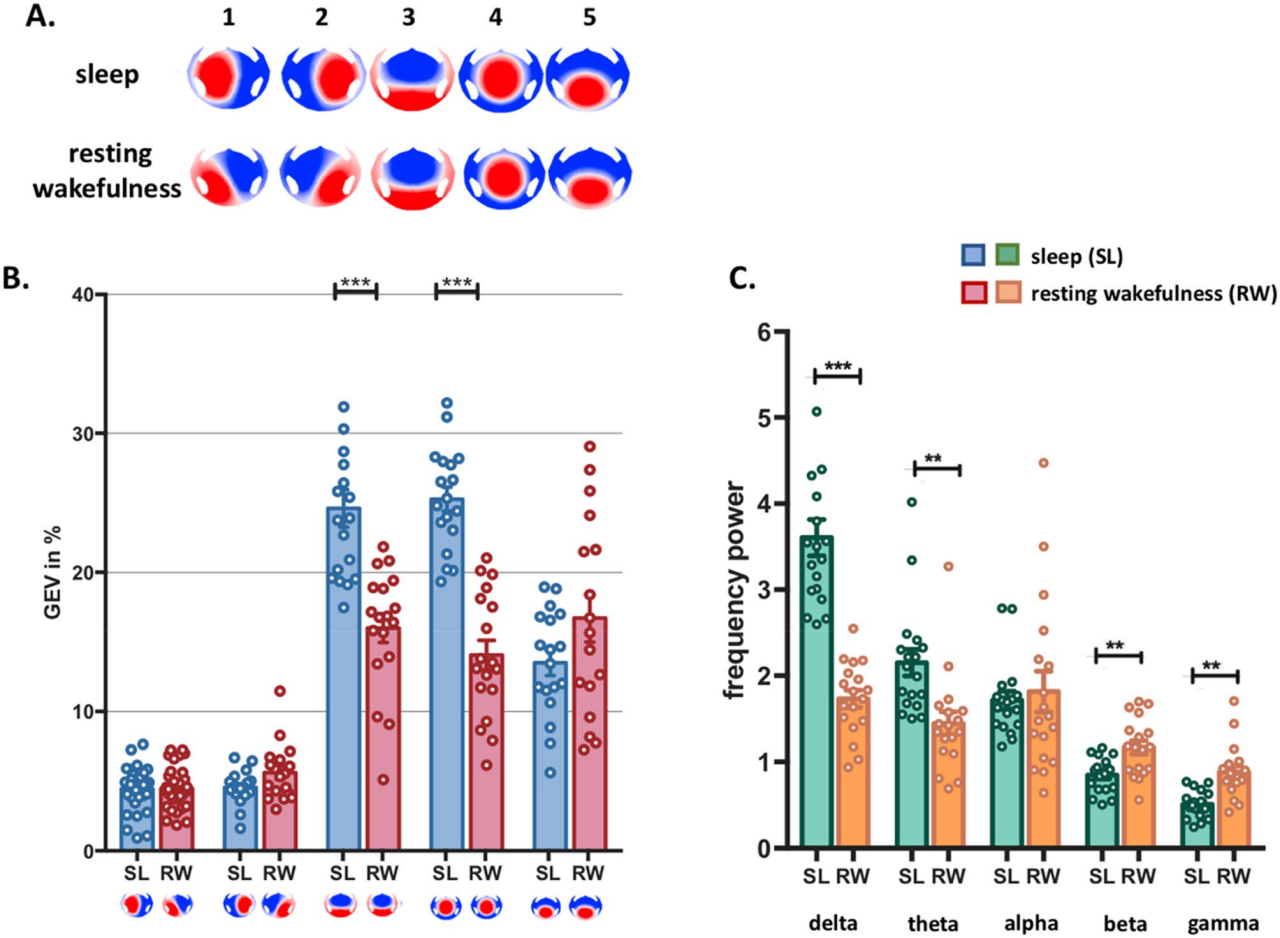
EEG microstates during NREM sleep vs. resting wakefulness. (**A**) The five EEG microstates identified by k-means cluster analysis across participants in the two conditions (NREM sleep and resting wakefulness). (**B**) The mean and standard error of the global explained variance (GEV) of each microstate in both conditions. Microstate map 3 and map 4 explained significantly more variance in the NREM sleep condition compared to resting wakefulness. (**C**) Mean power in the five frequency bands averaged across all microstates. As expected, low-frequencies dominated during NREM sleep in all microstates, while high-frequency power was higher during wakefulness (see Supplementary Fig. S1 for each microstate separately). (***p < 0.0001, **p < 0.001 Bonferroni corrected).

Of 38 participants who were recorded with 256-channel EEG during a whole-night sleep, we first analyzed spontaneous EEG data in a subset of 18 participants in NREM sleep (SL) vs. resting wakefulness (RW). Throughout the night, participants were awakened^16^ and asked to report whether they had been dreaming (dreaming experience, DE) or not (no experience, NE). To identify microstate maps that might be specific to sleep as compared to awake, we concatenated 30 s epochs of SL before the awakenings, independent of whether participants reported dreams or not, and eight randomly selected artifact-free 30 s epochs of RW. A spatial *k*-means cluster analysis^19^ was applied to the concatenated EEG of each participant, separately for SL and RW. A meta-criterion^25^ revealed between 4–10 clusters optimally explaining the data of each participant. The best cluster maps of each participant were then used for a group cluster analysis across participants for each condition (SL and RW). The meta-criterion revealed five maps optimally explaining the SL as well as the RW groups each. The five microstate maps showed highly similar topographies in both groups (Fig. 1A), particularly concerning microstates 3–5, as indicated by spatial correlation analysis (Supplementary Table S1).

The five dominant maps of both SL and RW were then fitted back to the original EEG of each participant by spatial correlation analysis and winner-takes-all labelling. The fitting procedure allowed us to calculate the occurrence of each microstate, the duration during which the states were present without interruption, and the global variance explained by each of the dominant maps (GEV). These parameters are typically used to characterize the spatiotemporal properties of EEG microstates. The analysis of these parameters revealed that two microstates dominated the sleep EEG in terms of duration and global explained variance (GEV). While microstates 1 and 2 explained less than 5% of the global variance, and map 5 on average 14%, microstate 3 and microstate 4 together explained nearly 50% of the variance (map 3: 24.61%, map 4: 25.27%) (Fig. 1B). Crucially, microstates 3 and 4 explained only 32% of the variance during resting wake (map 3: 18.18%, map 4: 14.22%), indicating that their presence is markedly different between sleep and wakefulness [2 × 5 repeated measures ANOVA interaction: F(2.08, 35.36) = 21.78, p < 0.0001, η^2^ = 0.56, ε = 0.52; ANOVA microstate 3: F(1, 17) = 27.15, p < 0.0001, η^2^ = 0.61; microstate 4: F(1, 17) = 96.67, p < 0.0001, η^2^ = 0.85]. Maps 3 and 4 also lasted longer than the other microstates during SL vs. RW. [2 × 5 repeated measures ANOVA interaction: F(1.9, 32.4) = 67.88, p < 0.0001, η^2^ = 0.8, ε = 0.47; ANOVA microstate 3: F(1, 17) = 14.49, p = 0.001, η^2^ = 0.46; microstate 4: F(1, 17) = 23.0, p < 0.0001, η^2^ = 0.57].

To determine the spectral correlates of each microstate we performed a time–frequency analysis of the original EEG of each subject and calculated the average power in the different frequency bands separately for all time points labelled with a given microstate. The mean power was then averaged across all electrodes. As expected, during NREM sleep all microstates were dominated by low-frequency power, while during wakefulness, the higher frequencies dominated (Fig. 1C) (see Supplementary Fig. S1 for details). Thus, while the topographies of the microstates remain the same, the frequency in which these microstates temporally synchronize differ between sleep and wakefulness. Interestingly, microstates 3 and 4, the ones dominating in sleep also show higher low-frequency power than the other microstates (Supplementary Fig. S1). This supports the hypothesis that the networks generating these two microstates are most inhibited during NREM sleep.

In sum, while the microstate topographies that are independent of the EEG frequency content appear similar during sleep and rest, the time during which these microstates were present and the amount of variance they explained differed significantly. We then asked whether the microstates reflect distinct functional states of the sleeping brain, that is, whether they are differently related to dreaming experiences during sleep.

### Dissociation of EEG microstates during dreaming experience vs. no experience

**Figure 2:**
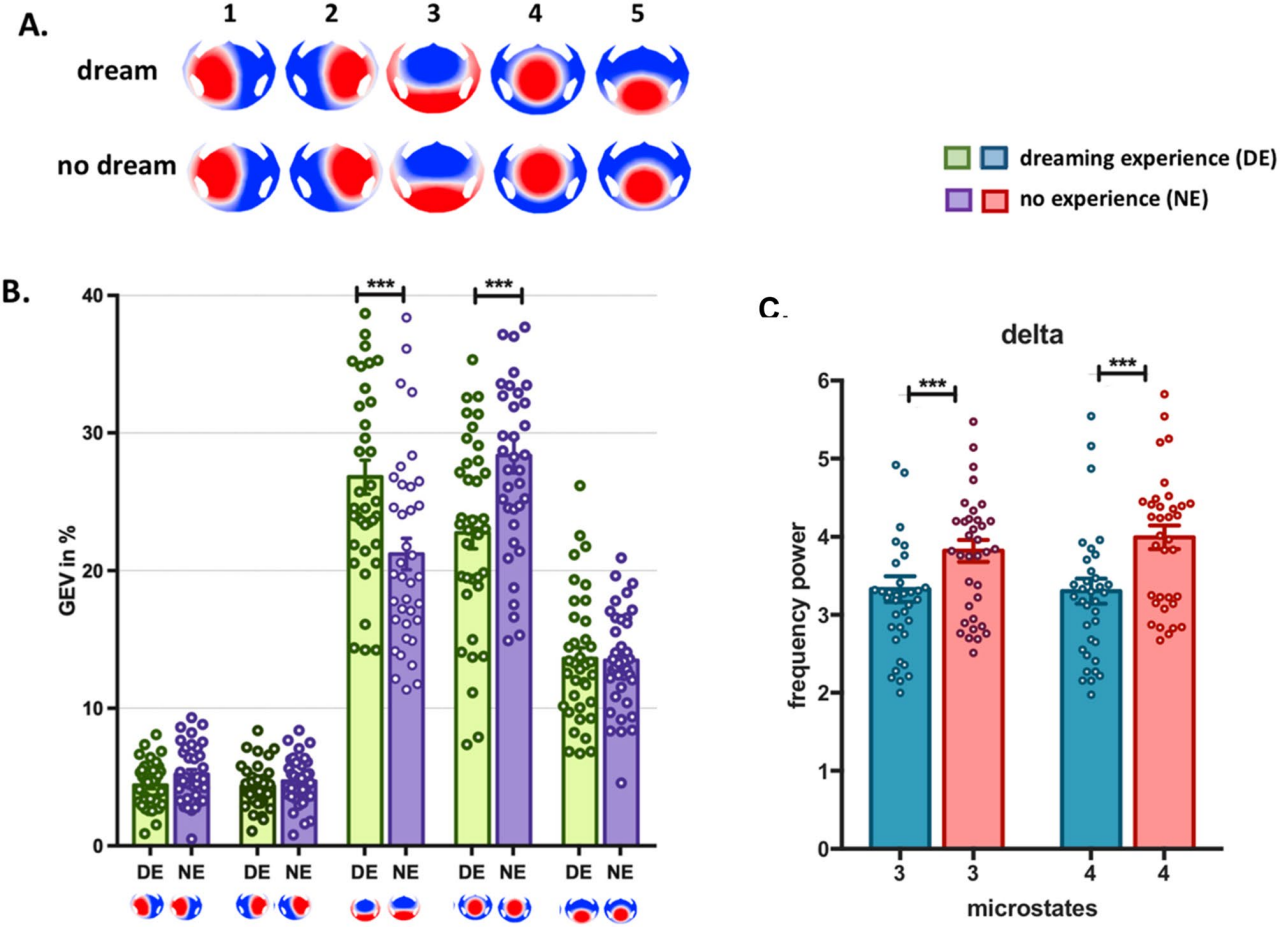
Double dissociation in EEG microstates during dreaming experience vs. no experience. (**A**) The five EEG microstates identified by *k*-means cluster analysis across participants in two conditions (dreaming experience, DE and no experience, NE). (**B**) The mean and standard error of the global explained variance (GEV) of each microstate in both conditions. Microstate 3 explained significantly more variance in the conscious experience condition (DE), while microstate 4 explained significantly more variance in the no conscious experience condition (NE). (**C**) Mean power in the delta frequency band for microstate 3 and microstate 4 in DE and NE. Delta power decreases significantly when dreams are recalled for both microstates (for details of all microstates see Suppl. Fig. S2). (***p < 0.0001).

In the second step, we compared the temporal characteristics of the microstates between dreaming and no dreaming experiences within NREM sleep. To do so, we identified the most dominant EEG microstate maps for DE and NE separately. We analyzed the whole set of 38 participants (note than one participant was excluded due to no DE report). The meta-criterion determined five optimal EEG map clusters for each condition (Fig. 2A). The topographies of these five maps were again strikingly similar between the two conditions (for the spatial correlation see Supplementary Table S2) and identical to the five maps found in the subset of 18 participants reported in the prior sleep vs. rest analysis.

Next, we fitted the maps back to the original EEG of each participant to determine the global explained variance, the mean duration and the occurrence of each microstate. We found that the two microstate maps that dominated in sleep vs. rest (map 3 and map 4) also significantly differed in their presence between DE and NE. Map 3 explained significantly more variance in the DE condition, while map 4 explained significantly more variance in the NE condition (Fig. 2B) [2 × 5 repeated measures ANOVA interaction: F(2.22, 80.04) = 69.13, p < 0.0001, η^2^ = 0.65, ε = 0.55; ANOVA microstate 3: F(1, 36) = 25.91, p < 0.0001, η^2^ = 0.41; microstate 4: F(1, 36) = 65.30, p < 0.0001, η^2^ = 0.64]. This dissociation was also significant in terms of duration [2 × 5 repeated measures ANOVA interaction: F(2.92, 105.42) = 73.73, p < 0.0001, η^2^ = 0.67, ε = 0.73; ANOVA microstate 3: F(1, 36) = 10.48, p = 0.003, η^2^ = 0.22; microstate 4: F(1, 36) = 12.01, p = 0.001, η^2^ = 0.25). In addition, the occurrence of map 3 was significantly higher in DE vs. NE (F(1, 36) = 12.51, p = 0.001, η^2^ = 0.25)]. We then compared the frequency power of each microstate between DE and NE to test whether the observed double dissociation between microstates 3 and 4 was due to differences in low-frequency power between these states. As expected from previous studies, we found a general decrease of low-frequency power and an increase of high-frequency power during DE compared to NE for all microstates (see Supplementary Fig. S2). Figure 2C shows this result for microstates 3 and 4 and illustrates that the double dissociation found in the temporal parameters of microstates 3 and 4 are not explained by dissociations in the frequency content of the slow-wave activity of these microstates.

Having identified two brain microstates that dominate during NREM sleep compared to resting wakefulness, but dissociate within NREM sleep between dreaming and no dreaming experiences, we then investigated which brain regions are generating these EEG microstates.

### Two distinct brain networks dissociate dreaming from no dreaming during NREM sleep

**Figure 3:**
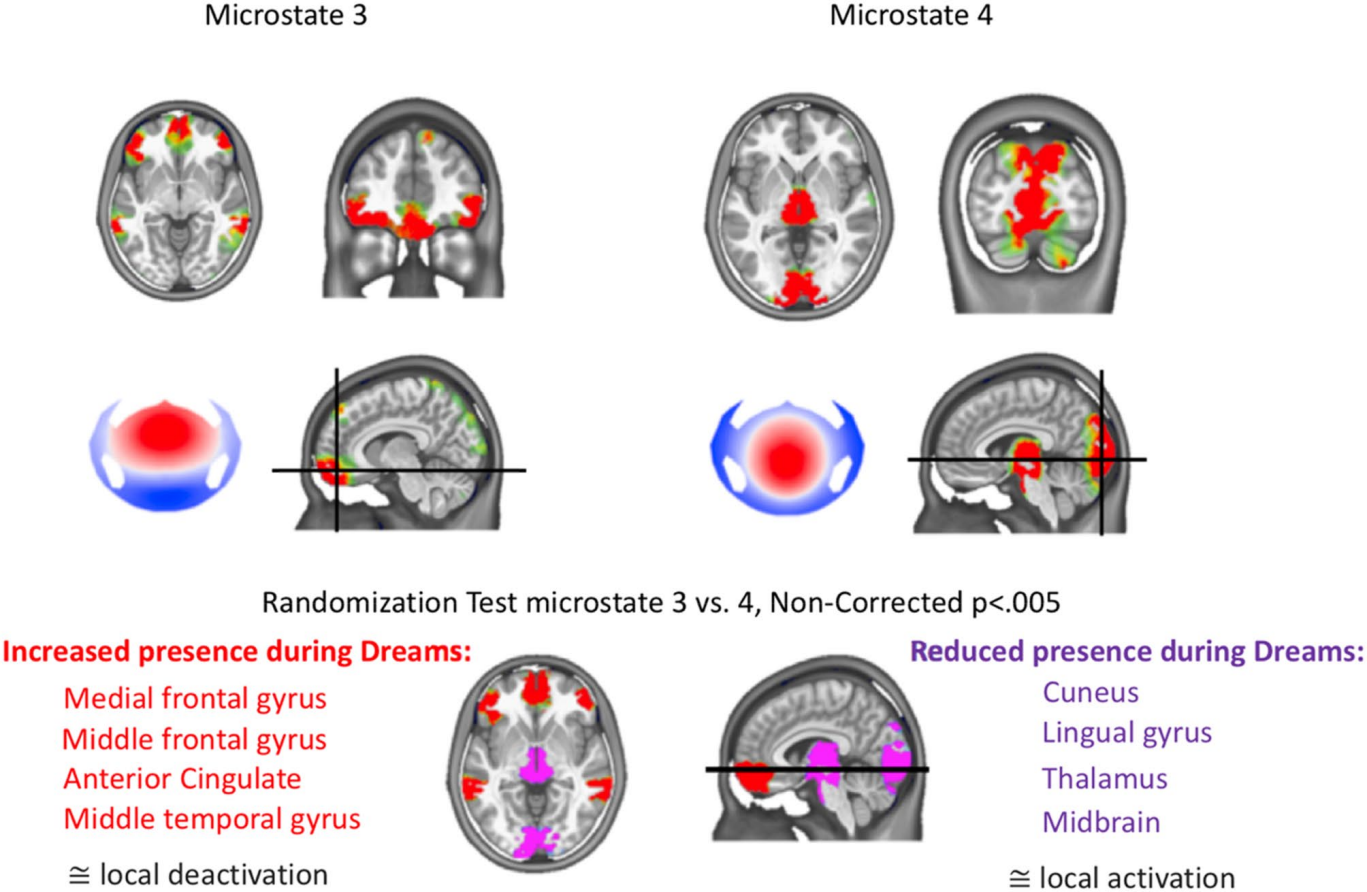
Two distinct brain networks dissociate dreaming experience from no experience during NREM sleep. (**A**) Microstate 3, which was dominant during DE, was mainly localized in the medial and medial frontal gyrus, the anterior cingulate and the middle temporal gyrus. (**B**) Microstate 4, which was reduced in DE compared to NE, was mainly localized in occipital brain areas (cuneus and lingual gyrus), but also in the thalamus extending to the midbrain of the brainstem. (**C**) Statistical comparison of the two source distributions of microstates 3 and 4 was compared by paired randomization tests in the source space across participants. There was significantly more involvement of the medial and middle frontal gyrus and the middle temporal gyrus for microstate 3 and of the occipital cortex and the thalamus for microstate 4. An increased presence of a microstate during NREM sleep is interpreted as increased local deactivation.

To define the brain networks of the microstates that were dominantly active during sleep and differed between dreaming and no dreaming (microstates 3 and 4), we performed 3D source analysis using a distributed linear inverse solution. This was done by concatenating all time points that were labelled with a given microstate map for each participant. We then calculated the sources for each time point and averaged the current densities across all time points^25^. As a result, we obtained an average source distribution for each microstate map of each participant and condition. The estimated source networks were highly different between the microstates 3 and 4 in the DE condition, as confirmed by paired randomization tests in the source space across participants. Microstate 3, which was dominant during DE, was mainly localized in the medial prefrontal cortex, while microstate 4, which was reduced in DE compared to NE, was mainly localized in occipital brain areas (Fig. 3, for a comprehensive illustration of the sources of all microstates see Supplementary Fig. S3, detailed images of the statistical comparison are given in Supplementary Fig. S4). Moreover, for microstate 4, we found contributions from the thalamus and the brainstem together with the posterior brain regions. Thus, we identified two very distinct networks, dynamically alternating over time, that were associated with dreaming: a frontal network that was more present and lasted longer when participants reported that they were dreaming, and a posterior network that was less present when participants were dreaming.

## Discussion

The main goal of this study was to determine whether there exist distinct transient brain states of the sleeping brain that may play a critical role in experiencing and remembering dreams during NREM sleep. A couple of previous studies suggested different brain regions and frequency modulations to be associated with the conscious experience during NREM sleep^13,16,17,26–28^. By using the EEG microstate approach, which identifies brain states by the spatial properties, rather than the spectral content of the EEG, we were able to identify and precisely time two microstates that dominate during sleep as compared to awake, and found that these two states dissociated the EEG during dreaming and no dreaming: microstate 3 was more present when participants reported their dreams, while microstate 4 dominated when no dreams were reported. We then successfully established that the brain networks activated during microstate 3 included mainly medial and middle prefrontal areas, while microstate 4 was generated predominantly by occipital areas as well as subcortical structures.

In Ref.^16^ the interpretation of activation of the posterior “hot zone” when dreaming was based on the fact that low-frequency power and slow waves decreased in these brain areas. Since low-frequency oscillations are traditionally associated with neuronal bistability during which network interactions are impaired^3,4^, the decrease of these rhythms is a sign of improved interaction and integration of the posterior brain areas, i.e. activation. Our results using the microstate analysis approach on the same data supports this finding: the sources of microstate 4 whose activity was reduced when dreams were reported, were located in occipital brain areas, similar to the sources in Ref.^16^ that showed reduced low-frequency power when dreams were recalled. Previous EEG microstate studies showed that microstate 4 (corresponding to microstate D in the literature) increases in duration and occurrence during attentional tasks compared to resting wakefulness^29^, and it has been suggested that this brain state reflects aspects of attention, re-orientation and focus switching^30^. Recently, Brechet et al.^25^ showed that microstate D increases in duration and occurrence when participants are asked to perform a mental arithmetic task during eyes-closed rest. In the same study, another microstate increased in duration and occurrence when participants were instructed to recall autobiographical episodes from their life, indicating that microstates are sensitive to the momentary mental activity. Based on the previous finding^16^ and the neural generators of microstate 4 in the present study, we thus speculate that the decrease in the appearance of microstate 4 during dreaming is due to a global reduction of synchronization in low-frequency oscillations in these brain regions (i.e. a desynchronization) and therefore to activation of “the posterior hot-zone”, which allows for visual experiences during dreaming. This interpretation would also explain why microstate 4 increases in duration and explained variance during NREM sleep in general as compared to awake: It would signify an increased deactivation of this “task-positive” network during sleep, impairing causal interactions between the nodes of this network. The analysis of the frequency content of the different microstates indeed showed that while all microstates were dominated by low-frequency power during sleep compared to wakefulness, this low-frequency-activity was strongest for microstates 3 and 4.

Following the same logic, the increase of microstate 3 during sleep compared to awake would likewise indicate an increased deactivation of this network. The sources of microstate 3 mainly include frontal brain regions, particularly the medial and middle prefrontal areas. It is generally recognized that frontal brain areas are deactivated during sleep^31–33^. Thus, the increased presence of microstate 3 during dreaming may reflect increased bistability and further “de-activation” of the frontal network. Increased bistability in prefrontal regions involved in executive control may thus counterbalance the activation of the occipital brain regions during dreaming, thereby preventing spontaneous awakenings and protecting the continuation of sleep. This interpretation is supported by the view that “frontal deactivation must play a key role in any neurocognitive theory of dreaming” (Ref.^34^, pp. 340–341), and that sleep involves “disengagement of prefrontal systems from posterior perceptual systems”^35^, pp. 533). A simultaneous EEG-fMRI study^36^ previously showed increased BOLD responses associated with slow waves and delta waves in the medial and middle pre-frontal areas very closely corresponding to the brain areas associated with microstate 3 in our study. This was interpreted as activation of the frontal cortex during dreaming. However, our findings suggest that the increased BOLD response in relation to low-frequency activity may reflect increased neuronal bistability, possibly triggered by subcortical inputs that lead to large type I slow waves^18^.

Instead of limiting the solution space to cortical structures or a-posteriori masking deep structures when describing the results as in Ref.^16^, we here report the source localization in the grey matter of the whole brain. We are aware of the limitations of EEG in sensing deep brain structures, even though recent reports with simultaneous intracranial and scalp recordings in humans^37,38^ indicated that such activity might spread to the scalp. The sources that we found in the thalamus and the midbrain of the brainstem related to microstate 4 deserve to be reported. The results give empirical support for subcortical evidence of dream generation reported earlier^39^. More specifically, some authors suggested a role of the thalamus in cortical processing of information during sleep^40^, while others described how phasic thalamocortical bursts may lead to vivid visual imagery during NREM dreaming^41,42^. As for the role of the brainstem in dreaming, it has been extensively discussed in the literature^39,43^. First Solms^15,44^ and then Perogamvros and Schwartz^43^ proposed that the dopaminergic reward circuits originating in the midbrain (ventral tegmental area) are related to dream generation.

In sum, in this study we show that dreaming in NREM sleep is determined by the spatial and temporal characteristics of two distinct EEG microstates. Dream experiences are more likely to occur when the presence of microstate 4 was significantly reduced, mainly involving brain areas in the occipital brain regions, the thalamus and the brainstem. However, this reduction of low-frequency synchronization in these regions is counterbalanced by an increased presence of microstate 3 and thus increased low-frequency synchronization in frontal brain regions, possibly preventing the brain from waking up.

The double dissociation between microstate 3 and 4 in terms of their temporal characteristics was not merely due to a differential association with low-frequency activity. The analysis of the spectral content of each microstate showed that the low frequency power during dream recall compared to no recall was similar for both of them. Thus, the microstate approach adds information that goes above and beyond to a mere frequency analysis.

## Methods

### Participants

38 healthy participants (14 males, age 44 ± 13.3 years (mean ± SD), range 24–65 years) were included in the study. The data of 32 out of the 38 participants are the same as those used for the analyses of experiment 1 reported in Siclari et al. (2017). We excluded 1 participant due to no DE report, resulting in a final analyzed sample of 37 participants in both conditions: dreaming experience (DE) vs. no dreaming experience (NE). Out of the 37 participants, a subset of 18 participants [6 males, age 45 ± 11.9 (mean ± SD), range 26–63 years] was used for the analysis of sleep (SL) vs. rest (RW). Thirteen out of these 18 participants were reported in Ref.^27^. Our sample sizes were similar to previous studies, which examined sleep using high-density EEG recordings^45,46^.

### Protocol

Awakenings in the sleep laboratory were performed at pseudorandom intervals, during N2 sleep, using a computerized sound of 1.5 s, administered through E-Prime (Psychology Software Tools, Pittsburgh, PA, USA). The awakenings happened at intervals of at least 20 min and participants had to be asleep for a minimum of 10 min. Before triggering any awakening, participants had to be in a stable sleep stage for a minimum of 5 min. Upon awakening, participants were asked to signal that they had heard the sound and then to report whether, just before the awakening, they were dreaming of anything (DE) or not (NE). If they had a DE, they were asked to describe its recent content (“the last thing going through your mind before the alarm sound”) and then underwent a structured interview with additional questions related to the content. On average there were 6 awakenings per subject (range 3–11) with a total of 119 DE and 106 NE. Almost half of the awakenings (N = 116) took place in the first part of the night, while the rest (N = 109) took place in the second half of the night. More detail about the procedure can be found in Ref.^16^.

### EEG recordings

Sleep and rest recordings were performed at the University of Wisconsin (Wisconsin Institute for Sleep and Consciousness) using a 256-channel high-density EEG (hd-EEG) system (Electrical Geodesics). Sleep scoring was performed over 30 s epochs based on standard criteria^47^. The EEG signal was sampled at 500 Hz and band-passed filtered between 1 and 50 Hz for both NREM and awake recordings. The EEG data was then down-sampled to 250 Hz, bad electrodes were interpolated using 3-D spherical splines and re-computed to the average reference. Independent component analysis (ICA) was performed to remove ocular, muscular and electrocardiographic artifacts using EEGLAB routines.

### EEG k-means clustering

The free academic software Cartool was used for the microstate analysis^48^, (https://sites.google.com/site/cartoolcommunity/). K-means clustering^19^ was applied to the data of each participant and each condition. Maps at local maxima of the Global Field Power (GFP), i.e. the time points of highest signal-to-noise ratio were used for the cluster analysis^49^. The polarity of the maps was ignored as inverted polarities appear due to oscillations of the same underlying generators^22^. Cluster-analysis was first used on the data of each participant and condition (SL, AW, DE and NE) then on the cluster maps derived from each participant within a condition.

To determine the optimal number of clusters in the within- as well as the across subject cluster analysis, 7 different criteria were used and merged to define a meta-criterion as the median of all optimal numbers of clusters across all criteria^25,50^. The following 7 criteria were taken from Refs.^19,51–53^:

1. Gamma: An adaptation of Goodman and Kruskal, based on concordant vs. discordant clustered pairs.
2. Silhouettes: Evaluation of the consistency of each cluster through its goodness of fit.
3. Davies and Bouldin: A function of the sum of the ratio of within-cluster to between-cluster separation.
4. Point-Biserial: A point-biserial correlation calculated between the distance matrix and a binary cluster index.
5. Dunn: An evaluation of the goodness of separation of all clusters.
6. Krzanowski-Lai Index: A ratio of the relative difference of the within-clusters dispersion.
7. Cross-Validation: A modified version of the predictive residual variance.

The meta-criterion calculation is implemented in the free academic software Cartool (https://sites.google.com/site/cartoolcommunity/).

### Frequency analysis

To determine the spectral correlates of each microstate, we performed a time–frequency analysis of the original EEG of each subject and determined the average power in the different frequency bands for all time points labelled with a given microstate. The mean power was then averaged across all electrodes, resulting in mean spectral power for each microstate and each frequency band (Delta: 1–4 Hz; Theta: 4.5–8 Hz; Alpha: 8.5–12 Hz; Beta: 12.5–25 Hz; Gamma: 25.5–40 Hz). For the time–frequency analysis we used the Morlet wavelet transform function in Matlab as part of the Brainstorm software (https://neuroimage.usc.edu/brainstorm), with a frequency resolution of 0.5 Hz and an FWHM of base 2 logarithms of the target frequency.

### EEG source localization

To estimate the sources contributing to each of the microstate maps, we calculated a distributed linear inverse solution (LAURA)^54^. The lead field for the inverse solution was calculated for 257 electrode positions and the average brain of the Montreal Neurological Institute (MNI) in a grey matter constrained head model using the LSMAC head model with 5000 distributed solution points^55^. A standardization across time was applied for each solution point to eliminate activation biases^25,55^. The estimated current densities of each participant were averaged across all time points that were attributed to a given microstate in each condition. A statistical analysis evaluated whether the sources of the microstates differed between conditions. Similar to the conjunction analysis suggested for fMRI data^56^, we first thresholded each source map to the solution points that were above the 95th percentile of activation in the mean map across all subjects.

### Statistical analysis

To investigate differences in the temporal parameters of the microstates between conditions we performed 2 × 5 repeated measure ANOVAs with the factors “condition” (sleep, rest or dreaming experience, no dreaming experience, respectively) and microstate class. Significant ANOVA effects were explored by post-hoc tests using Bonferroni correction. To evaluate differences of the microstates in the source space we performed randomization tests for each voxel between the microstates (p < 0.005 uncorrected). Only solution points above the 95th percentile of activation in the mean map across all subjects were considered in these statistical tests.

### Ethics statement

Signed informed consent was obtained from all participants before the experiment. Ethical approval for the study and the protocol for sharing the anonymized data were obtained from the University of Wisconsin-Madison Institutional Review Board. The experiment was performed following relevant guidelines and regulations.

## Supplementary information

Supplementary Information.
