# Supplementary material for: EEG microstates of dreams
Source: Sci Rep. 2020 Oct 13;10:17069. doi: 10.1038/s41598-020-74075-z (PMC7553905; doi:10.1038/s41598-020-74075-z)
Supplement: Supplementary file 1 — Supplementary Information. [file 41598_2020_74075_MOESM1_ESM.pdf]

Supplementary Information for

EEG microstates of dreams

Lucie Bréchet[1,2], Denis Brunet[1,2], Lampros Perogamvros[3,4,5], Gulio Tononi[5], Christoph M. Michel[1,2]

[1] Functional Brain Mapping Laboratory, Fundamental Neuroscience Dept., University Geneva, Switzerland
[2] Biomedical Imaging Research Center (CIBM), Lausanne, Geneva, Switzerland
[3] Sleep and Cognition Neuroimaging Laboratory, Fundamental Neuroscience Dept., University Geneva, Switzerland
[4] Sleep Laboratory, Division of Pneumology, Department of Medicine, Geneva University Hospitals, Rue Gabrielle- Perret Gentil 4, 1205 Geneva, Switzerland
[5] Wisconsin Institute for Sleep and Consciousness, Department of Psychiatry, University of Wisconsin - Madison, 6001 Research Park Blvd Madison, Wisconsin, USA

This PDF file includes:

Figures S1 to S2
Tables S1 to S2

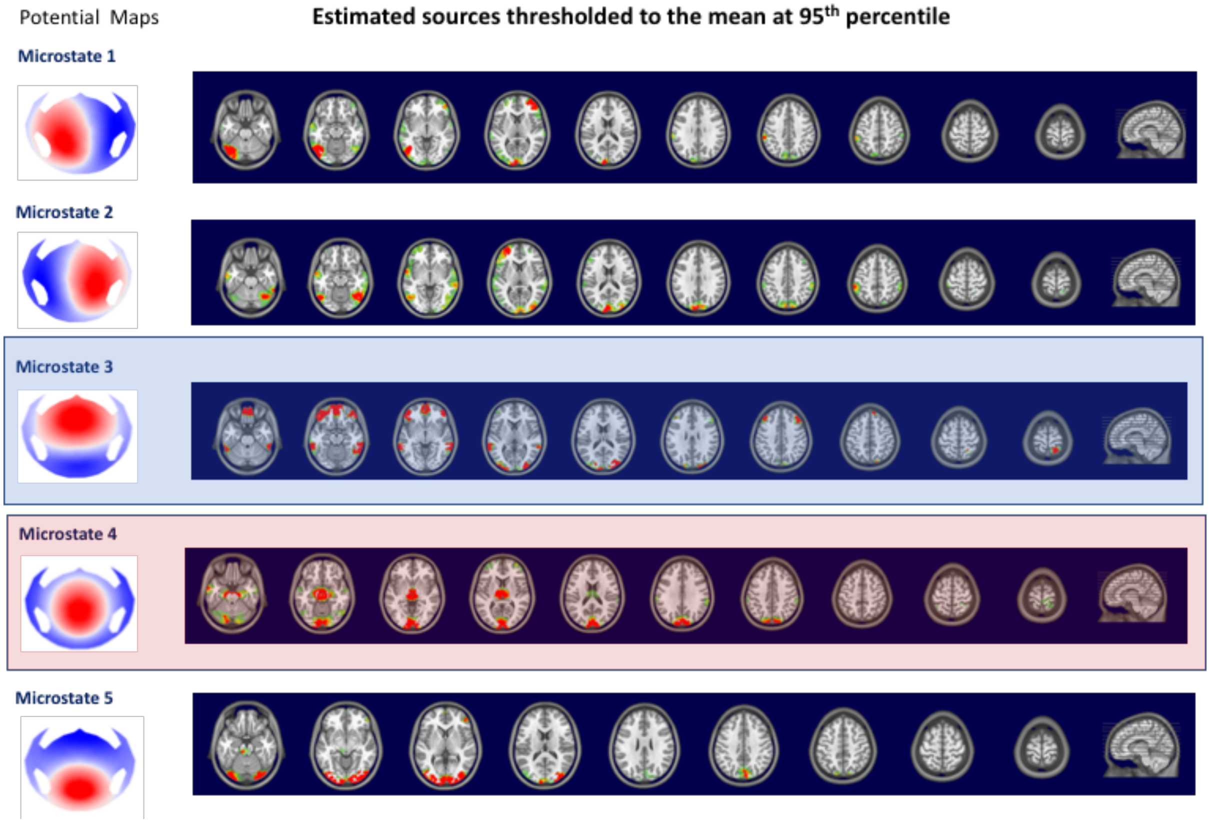

Fig. S1. Estimated sources of the five microstates during dreaming. *A distributed linear inverse solution (Laura) using the MNI template brain was applied to each time point that was labelled with a given microstate. The sources were normalized by the mean activity over the whole EEG for each solution point. The source maps were then averaged across all time points for each microstate and thresholded at the 95th percentile. The figure shows the average across the 37 subjects.*

Randomization Test microstate 3 vs. 4, DE condition. Non-corrected p< .005

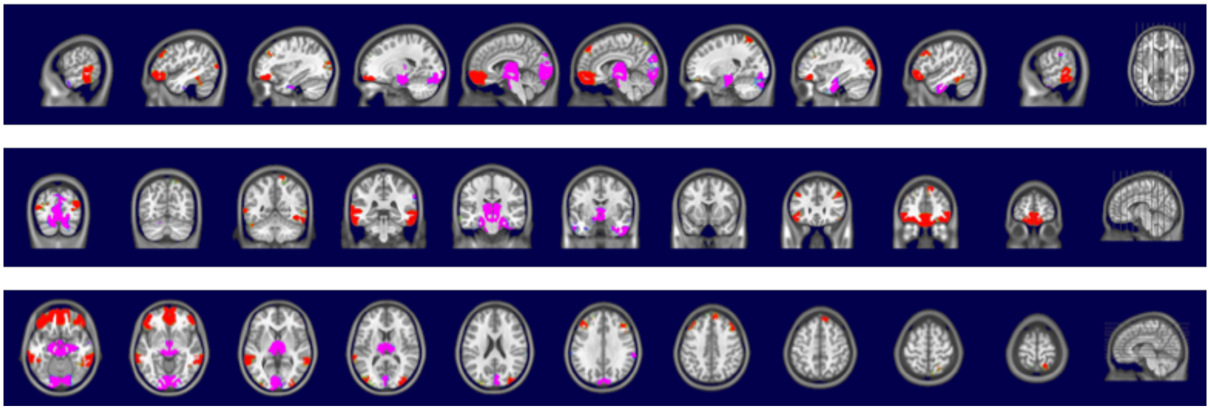

Fig. S2. Statistical comparison of the sources of microstates 3 and 4 during dreaming. ***Paired randomization tests were performed for each solution point (uncorrected $p<.005$). Red indicates areas that were more active when microstate 3 was present, and violet the areas more active when microstate 4 was present.***

		resting wakefulness				
		1	2	3	4	5
	DE/NE	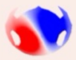	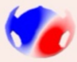	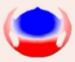	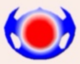	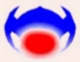
sleep 1	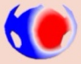	**0.73**	0.68	0.31	0.56	0.22
sleep 2	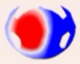	0.66	**0.70**	0.34	0.56	0.25
sleep 3	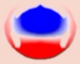	0.48	0.43	**0.98**	0.44	0.57
sleep 4	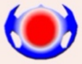	0.07	0.08	0.64	**0.91**	0.34
sleep 5	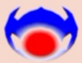	0.43	0.39	0.38	0.57	**0.98**

Table S1. Spatial correlation analysis between sleep and resting wakefulness. ***The five microstate maps revealed highly similar topographies in both groups as confirmed by spatial correlation analysis.***

		dreaming experience				
		1	2	3	4	5
	DE/NE	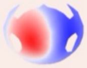	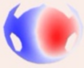	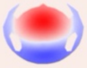	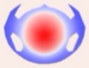	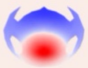
no experience 1	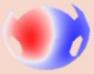	**0.96**	0.54	0.37	0.48	0.11
2	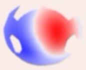	0.53	**0.96**	0.47	0.43	0.09
3	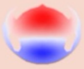	0.01	0.16	**0.94**	0.25	0.76
4	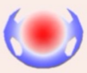	0.42	0.44	0.80	**0.93**	0.08
5	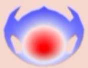	0.04	0.35	0.11	0.76	**0.90**

Table S2. Spatial correlation analysis between conscious experience and no experience. ***The topographies of these five maps were strikingly similar between the two conditions and identical to the five maps found in the subset of 18 participants reported in the prior sleep vs. rest analysis.***
